# Supplementary material for: Synthesis of Novel Tritopic Hydrazone Ligands: Spectroscopy, Biological Activity, DFT, and Molecular Docking Studies
Source: Molecules. 2022 Mar 2;27(5):1656. doi: 10.3390/molecules27051656 (PMC8911750; doi:10.3390/molecules27051656)
Supplement: Supplementary file 1 [file molecules-27-01656-s001.zip › molecules-1583723-supplementary.pdf]

Supplementary Materials

Synthesis of Novel Tritopic Hydrazone Ligands: Spectroscopy, Biological Activity, DFT, and Molecular Docking Studies

Sharmin Akther Rupa [1], Md. Rassel Moni [1], Md. Abdul Majed Patwary [1,*], Md. Mayez Mahmud [2], Md. Aminul Haque [3], Jamal Uddin [4,*] and S. M. Tareque Abedin [5,*]

1 Department of Chemistry, Comilla University, Cumilla 3506, Bangladesh; sharminrupa@cou.ac.bd (S.A.R.); monirassel@gmail.com (M.R.M.)
2 Faculty of Pharmaceutical Science, Tokushima University, Tokushima Shi 770-0026, Japan; hemmayez@yahoo.com (M.M.M.)
3 Department of Chemistry, Jagannath University, Dhaka 1100, Bangladesh; amin2k12@chem.jnu.ac.bd (M.A.H.)
4 Department of Natural Sciences, Center for Nanotechnology, Coppin State University, MD 21216, USA
5 Department of Chemistry, Jahangirnagar University, Savar 1342, Bangladesh
* Correspondence: mamajedp@gmail.com (M.A.M.P.); juddin@coppin.edu (J.U.); smtareque@juniv.edu (S.M.T.A.)

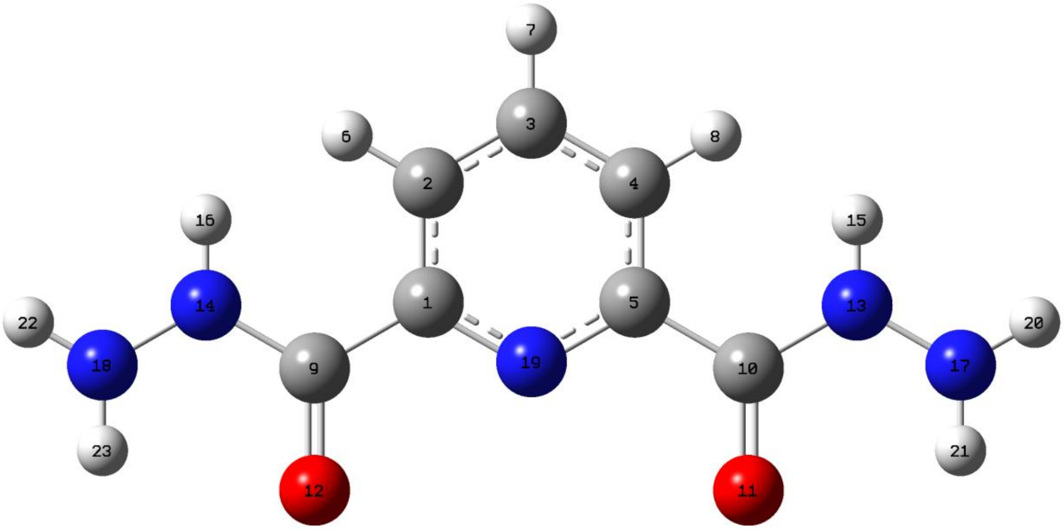

Figure S1. Optimized structures of the reactant 2,6-picolinic dihydrazone.

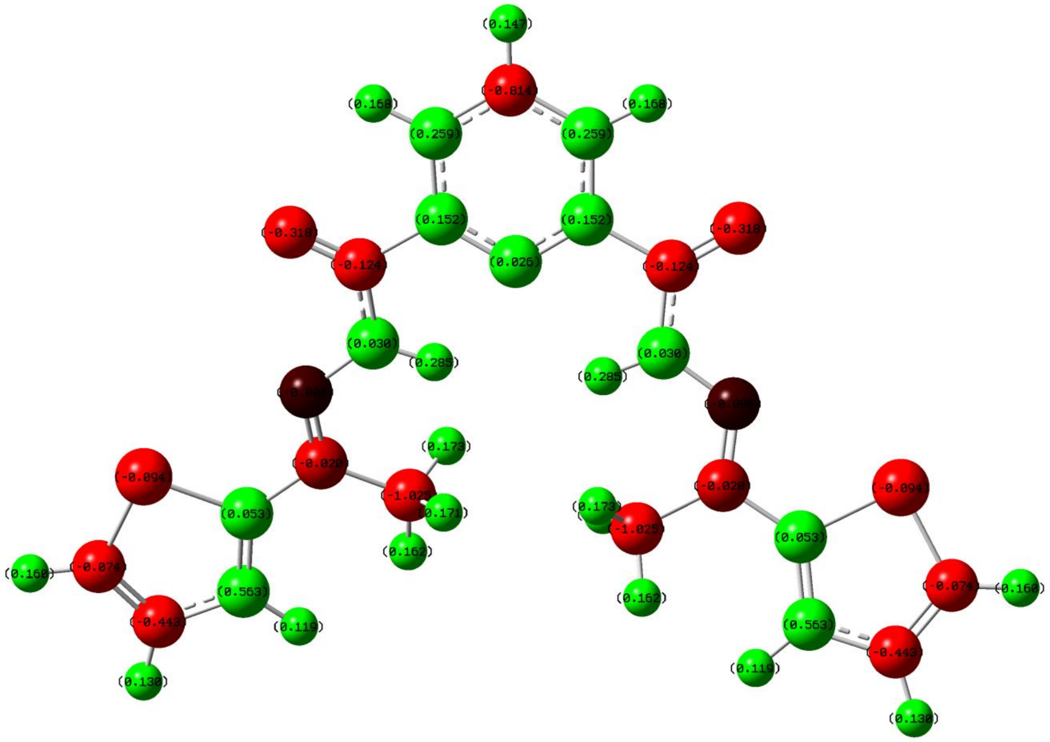

Figure S2. Partial atomic charge (Mulliken) on L1 where red represent negative, and green represent positive charge.

Table S1. Selected bond distances (Å) and bond angles (°) of reactant pyridine-2, 6-dicarboxylic dihydrazide.

Pyridine-2,6-dicarboxylic dihydrazide			
Atom position	Bond length (Å)	Atom position	Bond angle (°)
C(10)-O(11)	1.26	C(1)-C(9)-N(14)	120.00
N(13)-H(15)	1.00	O(11)-C(10)-N(13)	120.00
N(13)-N(17)	1.40	C(10)-N(13)-N(17)	119.99
L1			
C(10)-O(12)	1.21	C(1)-C(10)-N(14)	113.67
N(14)-H(45)	1.02	O(12)-C(10)-N(14)	124.87
N(14)-N(16)	1.36	C(10)-N(14)-N(16)	120.47
N(6)- C(1)	1.35	C(10)-N(14)-H(45)	116.90
S(43)-C(20)	1.75	H(45)- N(14)-N(16)	122.59
S(43)-C(24)	1.73	C(20)-S(43)-C(24)	91.40
L2			
C(10)-O(12)	1.22	C(1)-C(10)-N(14)	113.80
N(14)-H(43)	1.02	O(12)-C(10)-N(14)	124.78
N(14)-N(16)	1.36	C(10)-N(14)-N(16)	120.31
N(6)- C(1)	1.34	C(10)-N(14)-H(43)	117.14
N(45)-C(20)	1.38	H(43)-N(14)-N(16)	122.52
N(45)-C(24)	1.37	C(20)-N(45)-C(24)	110.18

Table S2. Theoretical and experimental ^{1}H and ^{13}C NMR chemical shifts (with respect to TMS, all values in ppm) for the title compounds.

**^{1}H NMR**					
**L1**			**L2**		
Atoms	δ_{exp} (in DMSO)	$\delta_{calc.}$ (in DMSO)	Atoms	δ_{exp} (in DMSO)	$\delta_{calc.}$ (in DMSO)
44-H, 45-H	11.43	10.77	42-H, 43-H	11.42	10.44
			46-H, 47-H	11.34	10.18
8-H, 9-H	8.27-8.31	8.85	8-H, 9-H	8.32-8.34	8.62
7-H	8.33-8.35	8.43	7-H	8.27-8.29	8.29
39-H, 41-H	7.66-7.67	7.77	39-H, 41-H	6.93-6.93	7.17
38-H, 40-H	7.14-7.16	7.30	38-H, 40-H	6.16-6.67	6.49
22-H, 27-H	7.61-7.62	7.66	22-H, 27-H		6.89
32-H, 36-H	2.52	2.31	32-H, 36-H	2.39	2.23
33-H, 37-H		2.31	33-H, 37-H		2.32
31-H, 35-H		2.79	31-H, 35-H		2.63
**^{13}C NMR**					
10-C, 11-C	159.44	163.51	10-C, 11-C	159.11	164.23
25-C, 20-C	154.55	156.44	25-C, 20-C	130.04	136.81
21-C, 26-C	129.44	133.13	21-C, 26-C	112.83	117.20
23-C, 28-C	128.20	131.47	23-C, 28-C	109.34	114.76
24-C, 29-C	130.06	140.65	24-C, 29-C	123.05	128.26
1-C, 5-C	148.91	155.99	1-C, 5-C	153.65	156.54
2-C, 4-C	125.75	131.21	2-C, 4-C	125.26	131.57
3-C	140.48	144.99	3-C	140.31	145.44
18-C, 19-C	143.22	154.35	18-C, 19-C	149.25	155.04
30-C, 34-C	15.32	12.36	30-C, 34-C	14.82	12.05

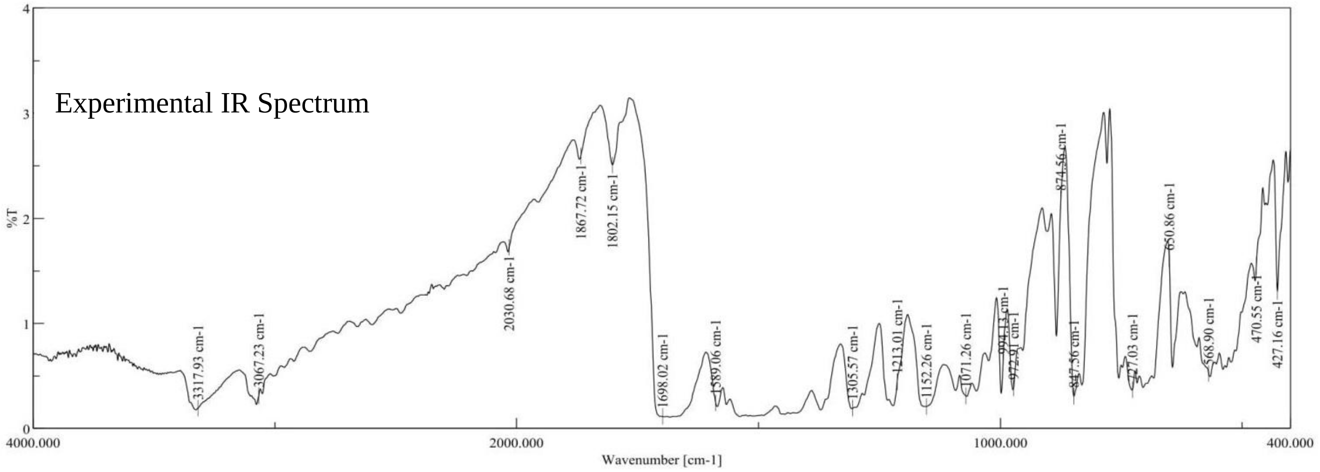

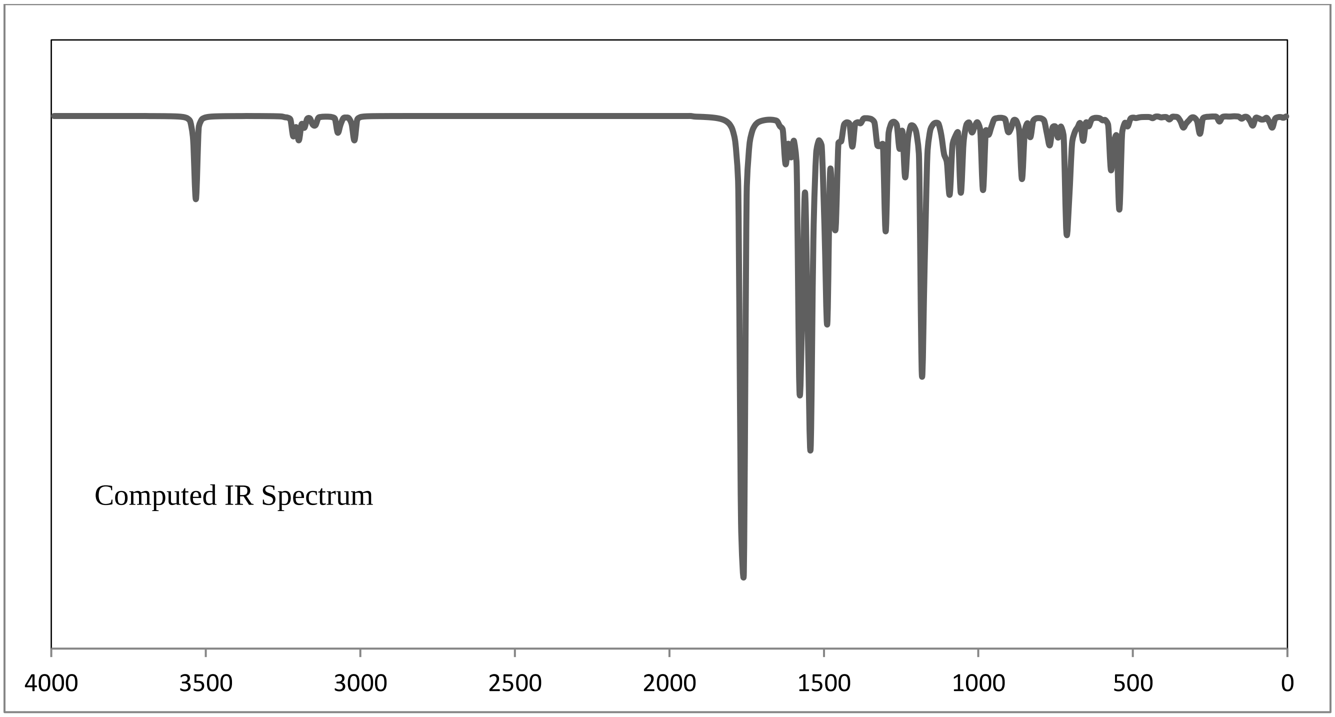

Figure S3. Experimental and computed Infrared Spectrum of **L1** ligand in KBr.

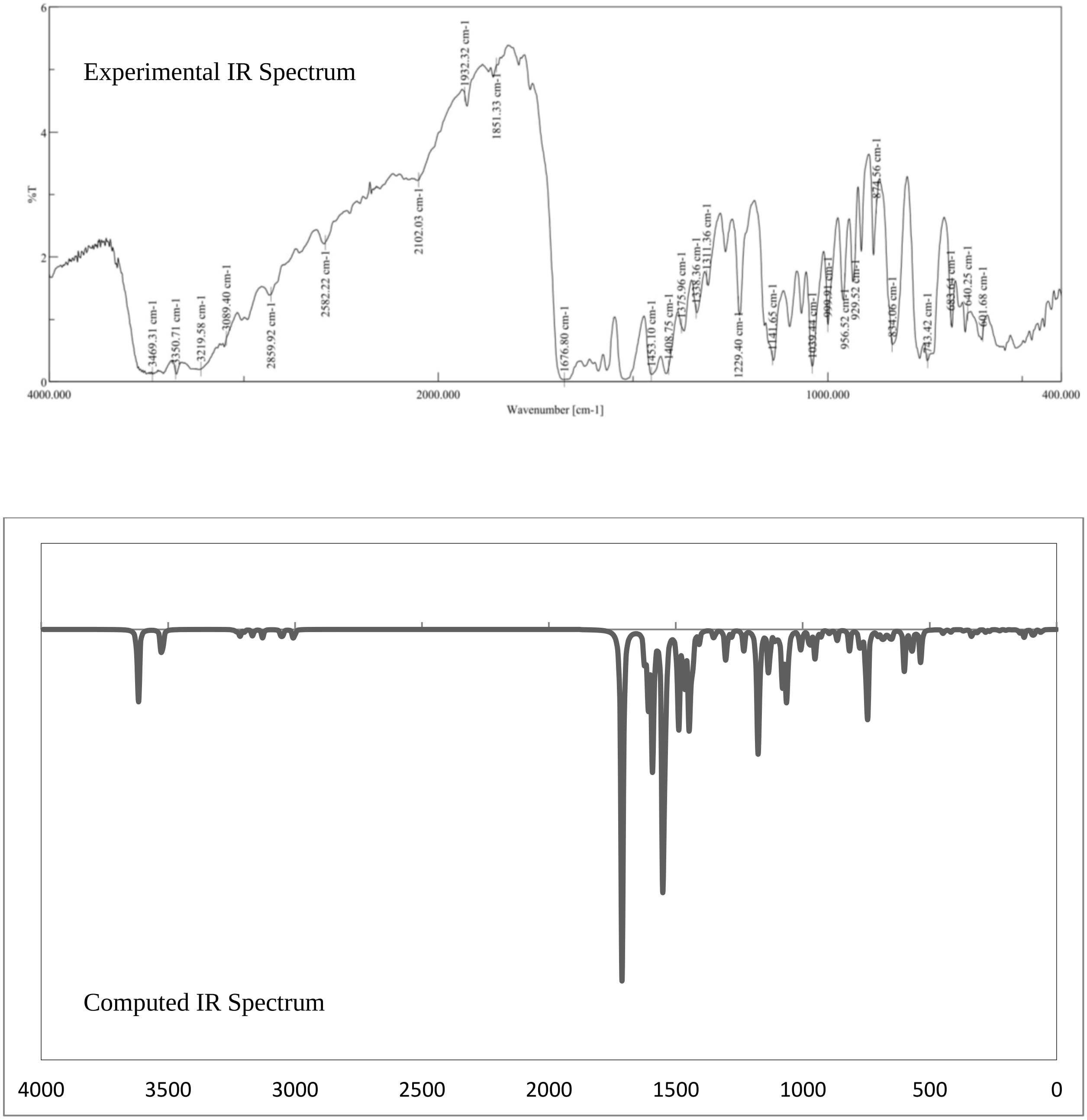

Figure S4. Experimental and computed Infrared Spectrum of **L2** ligand in KBr.

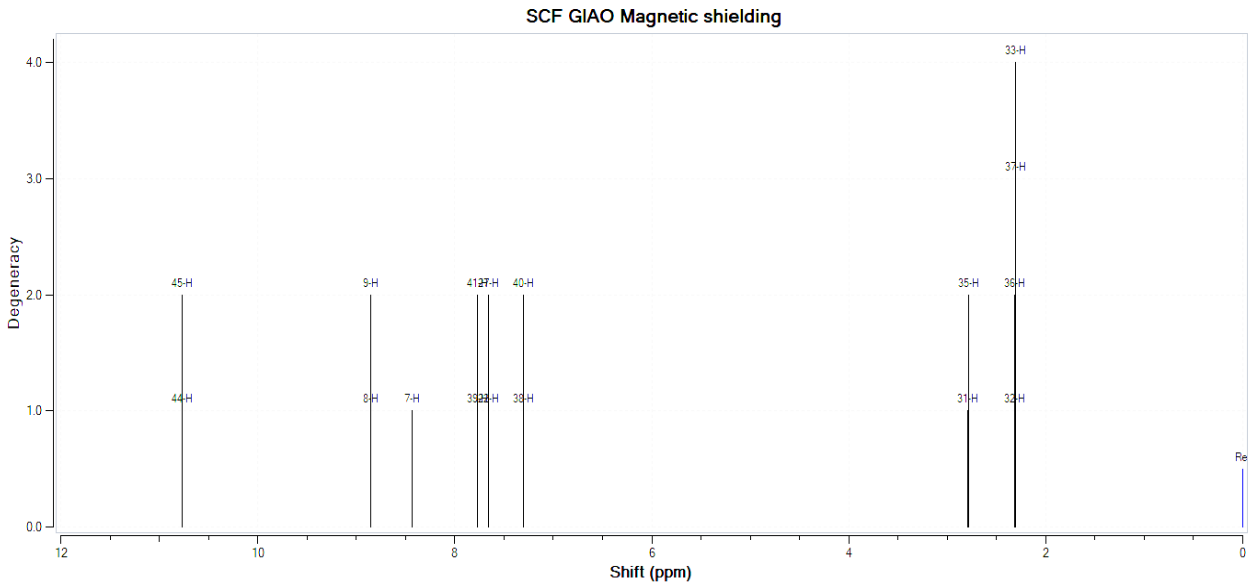

Figure S5. Computed ^{1}H NMR spectrum of **L1** in DMSO.

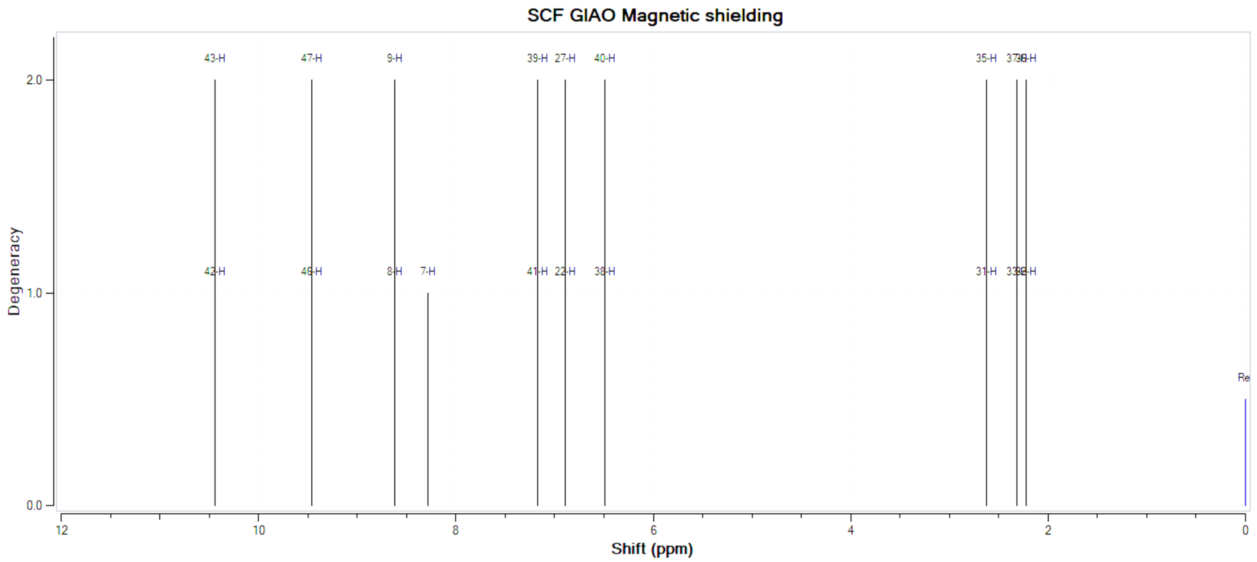

Figure S6. Computed ^{1}H NMR spectrum of **L2** in DMSO.

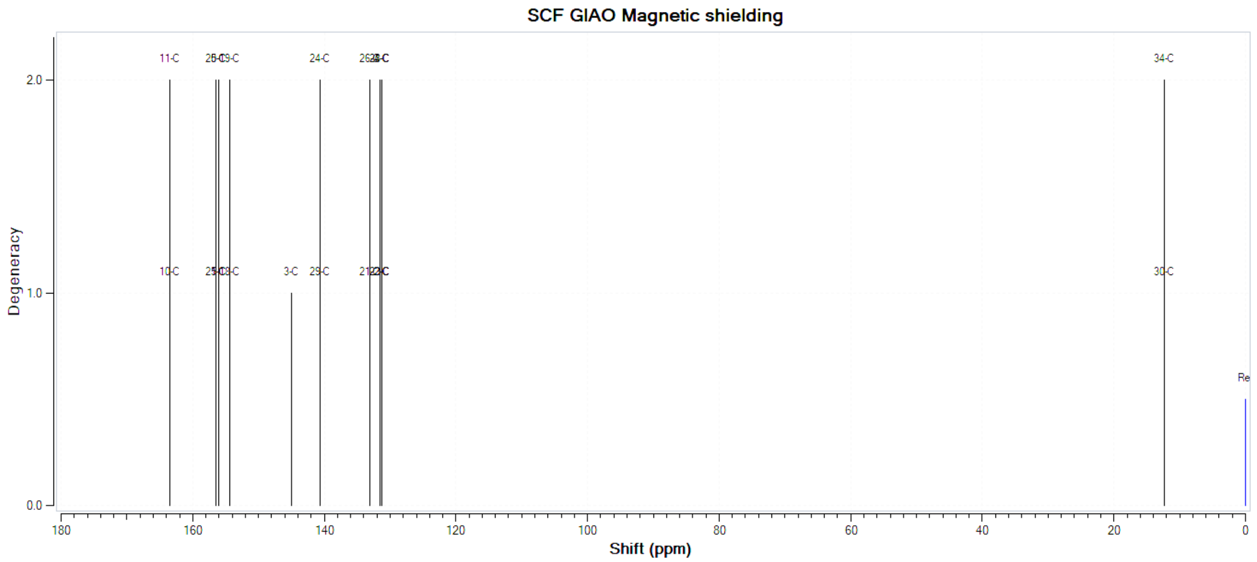

Figure S7. Computed ^{13}C NMR spectrum of **L1**.

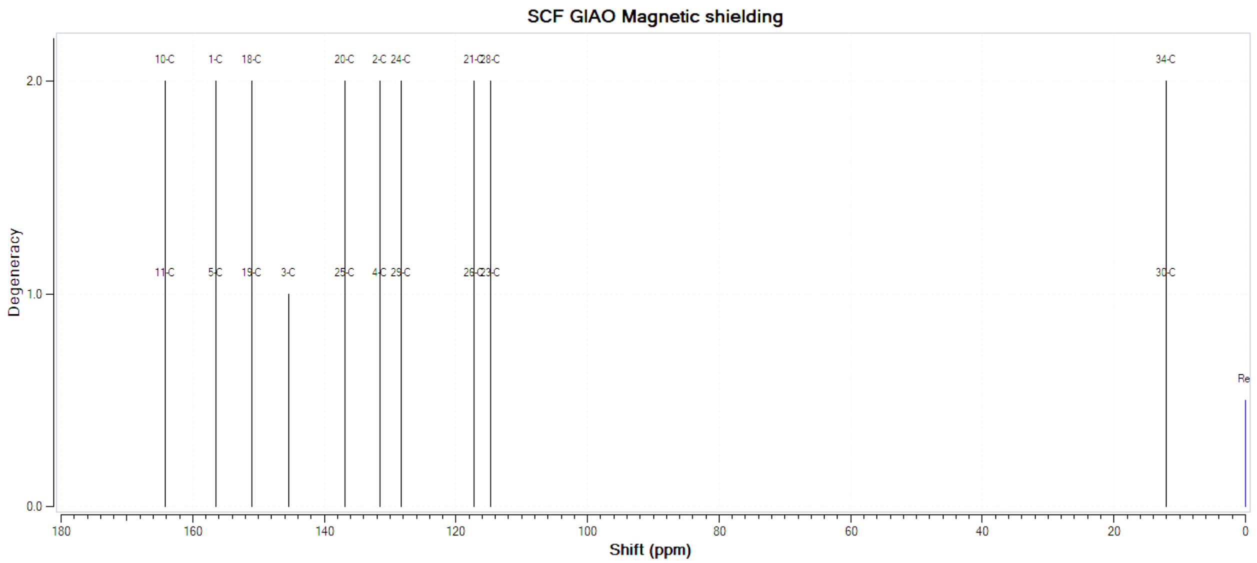

Figure S8. Computed ^{13}C NMR spectrum of **L2**.

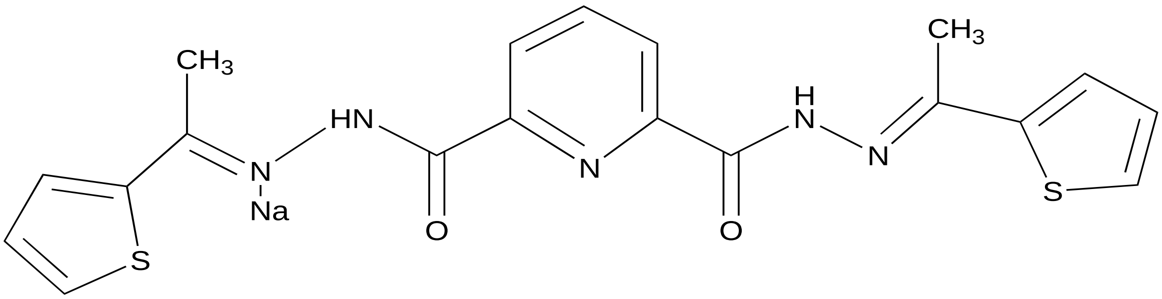

Chemical Formula: $C_{19}H_{17}N_5NaO_2S_2$
Exact Mass: 434.0721

Tolerance = 5000.0 mDa / DBE: min = -1.5, max = 50.0
Selected filters: None

Monoisotopic Mass, Even Electron Ions
1 formula(e) evaluated with 1 results within limits (up to 50 closest results for each mass)
Elements Used:
C: 19-19 H: 17-17 N: 5-5 O: 2-2 Na: 0-1 S: 2-2

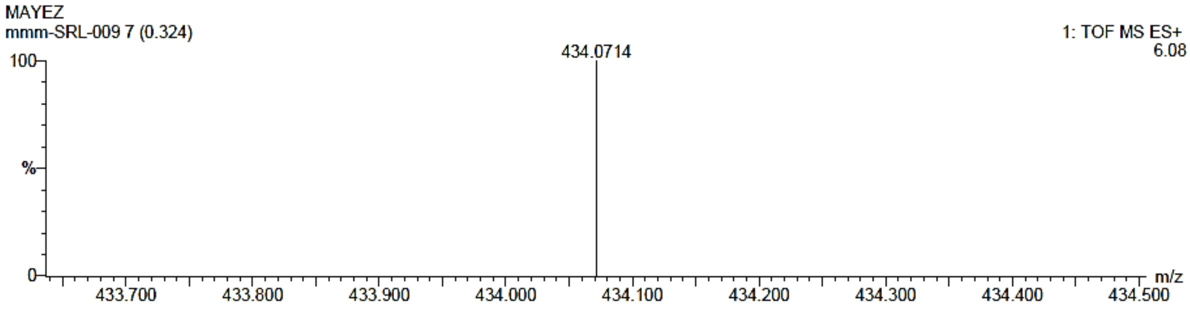

	Calc. Mass	mDa	PPM	DBE	i-FIT	Formula
Minimum:				-1.5		
Maximum:		5000.0	5.0	50.0		
Mass	Calc. Mass	mDa	PPM	DBE	i-FIT	Formula
434.0714	434.0721	-0.7	-1.6	13.5	451.2	C19 H17 N5 O2 Na S2

Figure S9. Mass spectrum of **L1**.

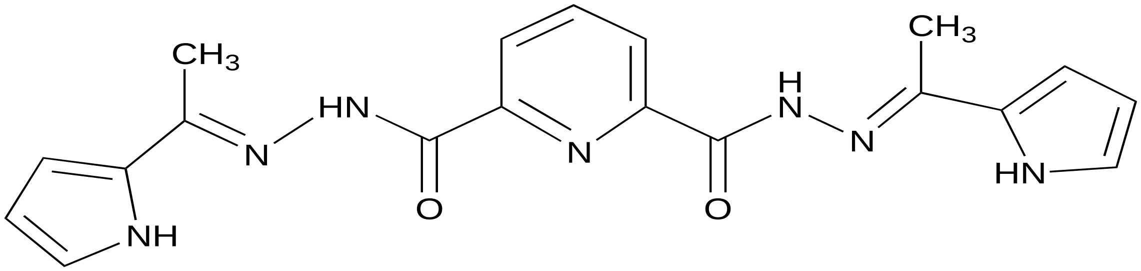

Chemical Formula: $C_{19}H_{19}N_7O_2$
Exact Mass: 377.16
Molecular Weight: 377.41

SRL-11

Tolerance = 5000.0 mDa / DBE: min = -1.5, max = 50.0
Selected filters: None

Monoisotopic Mass, Even Electron Ions
1 formula(e) evaluated with 1 results within limits (up to 50 closest results for each mass)
Elements Used:
C: 19-19 H: 19-19 N: 7-7 O: 2-2 Na: 0-1

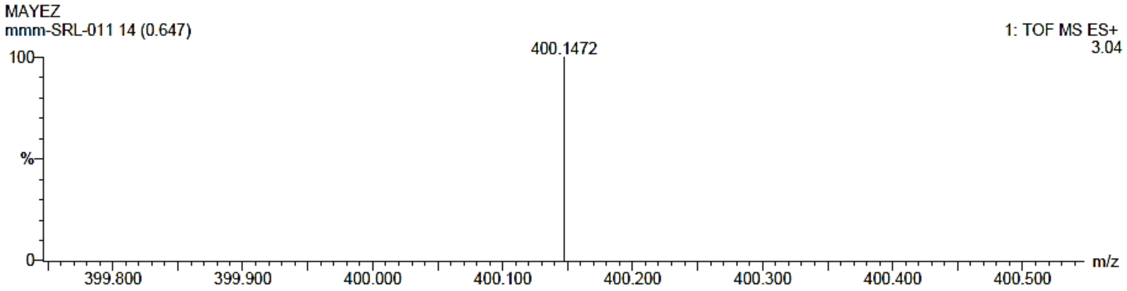

Minimum:				-1.5		
Maximum:		5000.0	5.0	50.0		
Mass	Calc. Mass	mDa	PPM	DBE	i-FIT	Formula
400.1472	400.1498	-2.6	-6.5	13.5	267.5	C19 H19 N7 O2 Na

Figure S10. Mass spectrum of **L2**.

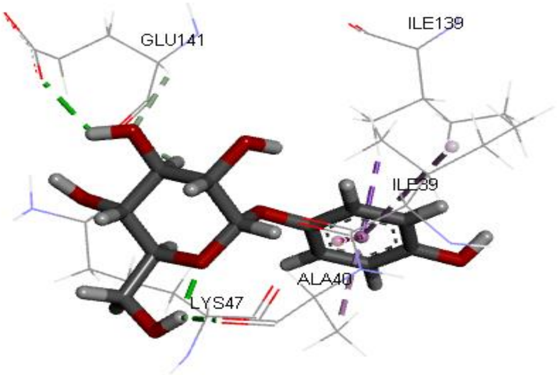

a) Arbutin-4j6u

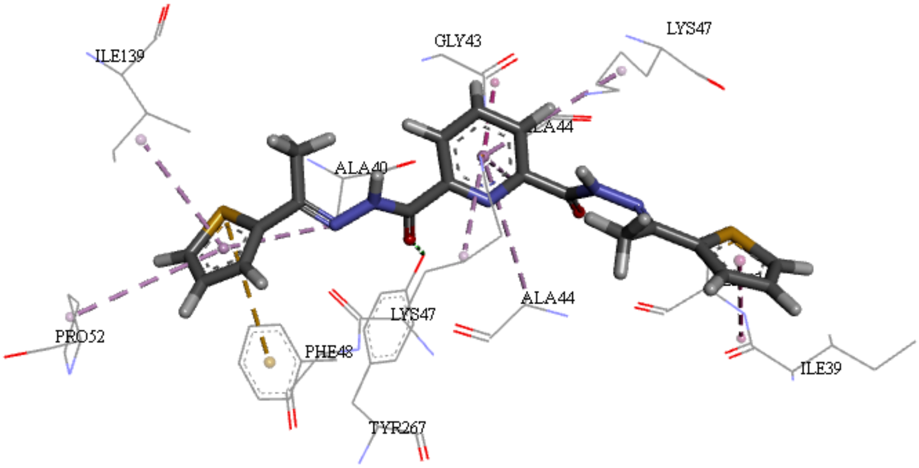

b) L1-4j6u

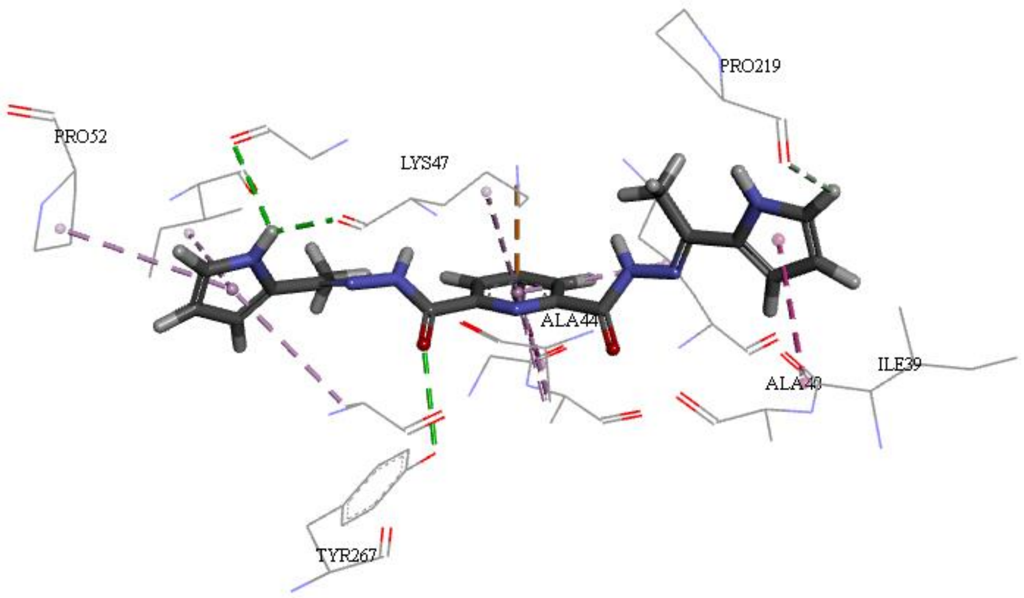

c) L2-4j6u

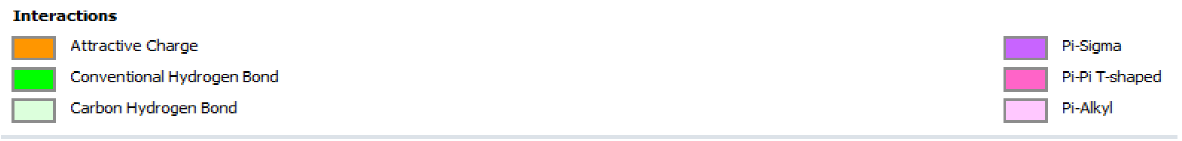

Figure S11. 3D Non-covalent interaction maps of a) Arbutin-4j6u b) L1-4j6u and c) L2-4j6u complexes.
